# A Deep Learning Framework With Domain Generalization and Few-Shot Learning for Locomotion Mode Classification Across Users, Sessions, and Prostheses

**DOI:** 10.1109/tmrb.2025.3606364

**Published:** 2025-09-04

**Authors:** Eugenio Anselmino, Ann M. Simon, Levi J. Hargrove

**Affiliations:** Department of Excellence in Robotics and AI, and The BioRobotics Institute, Scuola Superiore Sant’Anna, 56127 Pisa.; Shirley Ryan AbilityLab and the Department of Physical Medicine and Rehabilitation, Northwestern University Chicago, Evanston, IL 60208 USA.; Shirley Ryan AbilityLab and the Departments of Physical Medicine and Rehabilitation and Biomedical Engineering, Northwestern University Chicago, Evanston, IL 60208 USA.

**Keywords:** Locomotion mode classification, intention decoding, lower limb prostheses, deep learning

## Abstract

Transfemoral amputees don and doff their prostheses at least daily, making inter-session classification performance important for clinical implementation of locomotion mode classification algorithms. Here, we present a deep-learning framework based on domain-adversarial training and few-shot learning fine-tuning to classify locomotion modes in unseen sessions or subjects’ data across different prosthesis models. We validated the approach with a leave-one-session-out analysis repeated five times and made comparisons to a prosthesis-specific classifier. The dataset was created by merging data from two different prosthesis models (Vanderbilt University, VU, Gen 2 and Gen 3 powered knee-ankle prostheses), for a total of 31 sessions acquired across multiple days from 11 subjects. Subjects performed five locomotion tasks: level walking, incline and decline walking, and stair ascent and descent. Since transitions between different locomotion modes happen at different gait events, the analyses have been repeated for both heel-strike (HS) and toe-off (TO) events. At HS events, the proposed approach achieves a median f1-score of 99.12% and 92.41% on VU Gen 2 and Gen 3 prostheses respectively. At TO events, the proposed approach reaches a median f1-score of 96.83% with VU Gen 2 and 94.36% with VU Gen 3. The proposed framework is a promising solution for locomotion classification on data of previously unseen sessions or subjects, allowing classification on multiple prosthesis models.

## I. Introduction

LOWER limb amputations severely impact the quality of life of a person, reducing their independence and mobility [1]. Individuals with an amputation present a less efficient [2], slower, and less stable gait when compared to non-impaired people [3]. These deficits are often exacerbated during the execution of challenging tasks such as stairs and ramps ascent and descent [4].

Powered lower limb prostheses can improve amputees’ mobility due to the device’s ability to provide net mechanical energy, mimicking the biomechanical functionality of biological joints across different locomotion tasks [5], [6], [7], [8]. These devices effectively assist amputees with various movements, but the process of switching between modes is not seamless. Changing locomotion mode with a keyfob or via mechanical movements can help achieve reliable mode transitions, but it can be perceived as unnatural and cumbersome.

Machine learning (ML) and deep learning (DL) algorithms have shown promising results in locomotion mode classification. These algorithms predict users’ intended movements by interpreting prosthesis sensor signals [9], [10]. Both ML and DL approaches have achieved high classification accuracies in laboratory settings when trained on subject-specific data [10], [11]. However, these approaches are limited by their need for properly labelled training datasets from each end user. This limitation is particularly significant because achieving robust performance requires data collection across all desired locomotion activities and the transitions between them, which can be burdensome to collect [12].

To mitigate the limitations of needing large subjectdependent training datasets, domain-generalization (DG) and few-shot learning (FSL) strategies can be adopted. DG aims to learn from multiple related but distinct domains (e.g., subjects) in such a way that the model can generalize to a previously unseen domain (e.g., unseen sessions or subjects) [13]. FSL, instead, aims to maximize the learning with limited datasets by transforming the learning process into a similarity comparison with one or more data points from previously seen domains [14]. Recently, both DG and FSL approaches have gained popularity in upper-limb prosthesis control, especially with electromyographic (EMG) signals as they help mitigate EMG’s intrinsic subject dependency and temporal instability. DG approaches outperform traditional DL methods in cross-subject hand gesture classification, reaching accuracies similar to subject-specific approaches [15]. Similarly, DG-based DL models have improved the robustness of hand gesture classifiers across sessions held on different days with the same subject [16]. FSL approaches have shown comparable success, achieving convergence with only 20 samples from the target dataset [17] and outperforming traditional methods when dealing with limited datasets [18]

Here, we present a DL approach for transfemoral powered prostheses to classify five locomotion modalities: level walking (LW), stair ascending (SA), stair descending (SD), ramp ascending (RA), and ramp descending (RD). The approach uses signals from onboard prosthesis sensors and combines both DG and FSL methods to improve inter-session accuracy. We validated the approach using a dataset comprising 31 sessions from 11 different subjects, collected using two different prosthesis models: the Vanderbilt University (VU) 2nd and 3rd-generation powered knee-ankle prostheses [19].

## II. Materials and Methods

### A. Dataset

Detailed experimental protocols for data acquisition are described in [20] for the VU Gen 2 prosthesis and in [21] for the VU Gen 3 prosthesis. All subjects were unilateral transfemoral amputees and experienced prosthesis users. The subjects performed locomotion circuits including walking on level ground, ascending and descending a 10° incline, and ascending or descending a 5 or 6-step staircase. During level walking, subjects performed multiple starts and stops, turns, and shuffling to increase step variability. For both prosthesis models, an experimenter managed locomotion mode transitions using a key fob, with each transition occurring 90 ms after heel strike (HS), flat foot (FF), or toe-off (TO) events [22]. The possible transitions are shown in Table I.

The dataset comprises 24 acquisition sessions from 4 subjects using the VU Gen 2 prosthesis and 7 acquisition sessions from 7 subjects using the VU Gen 3 prosthesis. Data from both prostheses were merged into two single datasets, one for TO and one for HS events. FF events were excluded since the prosthesis always transitions from SA to LW, eliminating the need for classification. To enable DL models to work with both prosthesis generations, we selected only common signals between them: knee and ankle joint angles, velocities, and currents; leg anteroposterior, mediolateral, and vertical accelerations and angular velocities; shank and thigh angles; knee and ankle reference torques; and vertical load.

Signals from 210 ms before to 90 ms after the relevant gait events were selected and downsampled from 1 kHz to 200 Hz for computational efficiency. Information regarding dataset composition before the data augmentation and balancing processes is shown in Table II.

Before the model training phase, one session was removed from the dataset and used as test dataset. We adopted dataset augmentation techniques on the training dataset to reduce overfitting. We created 2 additional copies of the TO and HS datasets by temporally shifting the input window in a range of ±10 ms. We created 10 additional copies by scaling the original datasets and 8 additional copies by scaling the shifted datasets. During the scaling process, each channel was multiplied by a factor sampled from a uniform distribution between 0.95 and 1.05. Data have been normalized on a channel basis using robust scaling, with a scaling range between the 2^nd^ and 98^th^ percentiles.

To maintain balance during the training process, we selected 20,000 steps for each prosthesis model for SD, RA, and RD locomotion modes in the HS dataset. Since LW steps are more prevalent both in the dataset and in real-world scenarios, we selected 30,000 steps for the LW class for each prosthesis model. This approach helps retain more variability for the dominant class. Similarly, for the TO dataset, we selected 25,000 steps from the LW class and 20,000 from the SA class for each prosthesis model. If a class had fewer steps than the desired threshold, we generated additional copies by adding zero-centered Gaussian noise *(σ* = 0.025).

During the testing phase, data was scaled and normalized as per the training dataset. The fine-tune dataset is extracted from the test dataset according to the process presented in Section II-B2. Due to inter-session variability, the test dataset size and composition changed accordingly to the selected session. Nevertheless, more than 50 steps were always present for SD, RD, and RA classes and more than 90 for the SA class. The LW class was dominant in both TO and HS datasets, with more than 1000 steps.

### B. Classification Approach

We leveraged both DG and FSL approaches to develop a neural network capable of working on multiple prosthesis models and generalizing on previously unseen acquisition sessions or subjects. The training approach is based on two sequential steps: the first one leverages DG to pre-train the neural network; the second adopts FSL to fine-tune the network using a small portion of the test dataset. The process is repeated to create two distinct neural networks, one for the classification at HS and one for classification at TO.

#### 1) Pre-Training Network:

**Fig. 1.**
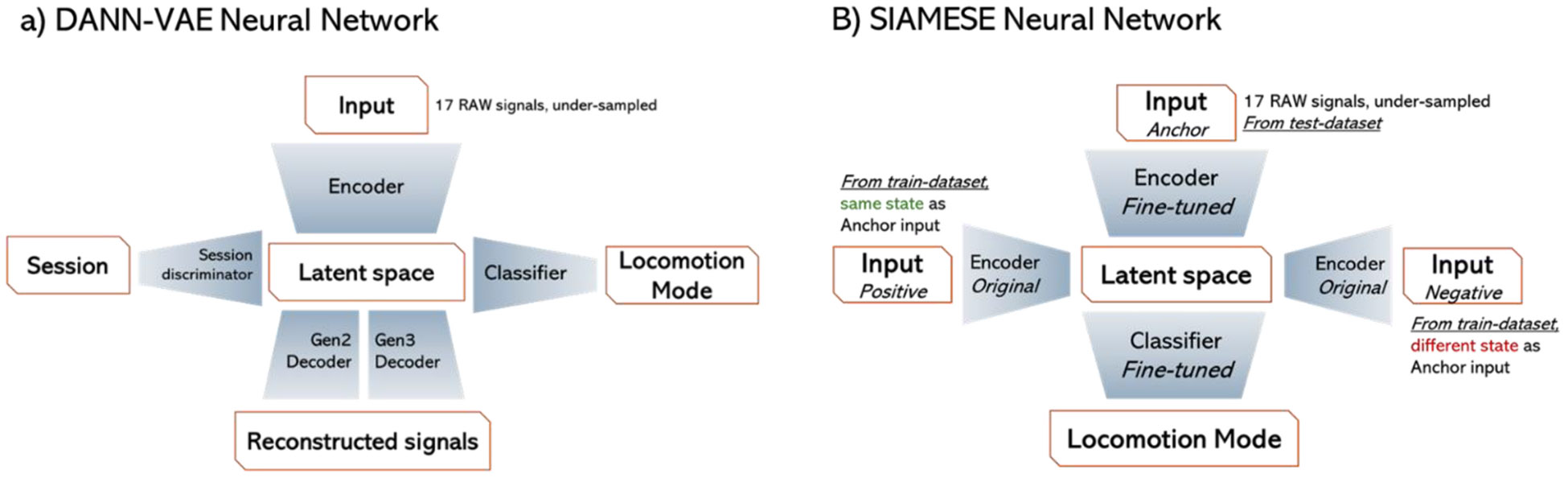
Overview of the neural network structure used for the pre-training (a) and fine-tuning (b) processes.

A neural network with a hybrid structure between Domain-Adversarial-Neural-Network (DANN) [23] and a Variational-Auto-Encoder (VAE) [24] was adopted to pretrain the network (Fig 1a.) The main objective of the DANN-VAE is to promote the Encoder sub-model to extract features from the input that allow the locomotion mode classification, via the Classifier sub-model, and the reconstruction of the input signals, via the Gen 2 and Gen 3 Decoders. Concurrently, the gradient from the Session discriminator is reversed to encourage the Encoder to extract session-independent features, as explained in [23].

The *Encoder sub-model* consists of an input layer for the time series, a corruptor layer that applies additive zerocentered Gaussian noise *(σ* =0.05), a sequence of three stride convolution layers with batch normalization and dropout, a flattening layer, and a latent encoder layer. In convolution layers, we used two-dimensional kernels to combine movement-related information across channels and temporal downsampling to encourage learning time-invariant features. Each convolutional layer used 256 filters, Leaky ReLU activations *(α*=0.2), and L2 normalization *(λ*=10-6). For the latent layer, we followed the implementation of [24], to map the extracted features to an 80-dimensional manifold.

The *Classifier and Session discriminator sub-models* have the same architecture. They consist of a corruptor layer *(σ* =0.05), a 64-neuron dense layer with batch normalization and Leaky ReLU activation *(α*=0.2), a dropout layer, and an output layer with softmax activation. Accordingly to [23], the loss of the Session discriminator is inverted for the training of the Encoder to promote the learning of session-invariant features.

Each *Decoder sub-model* consists of a corruptor layer *(σ* =0.05), a dense layer with batch normalization and Leaky ReLU activation *(α*=0.2), followed by a dropout layer and a reshape layer (features, 1, filters), and two stride transposed convolutional layers. The transposed convolutional layers are responsible for expanding the feature compressed by the Encoder to match the original input shape. Each transposed convolution layer uses 256 filters and two-dimensional kernels and is combined with batch normalization and dropout; the first layer uses a Leaky ReLU activation *(α*=0.2), and the second layer uses a Hyperbolic tangent activation.

The hyperparameters *σ* , *α*, and *λ* of the sub-models have been selected in accordance with [25].

#### 2) Fine-Tuning Network:

To fine-tune the Encoder and Classifier sub-models, five samples from each prosthesis state are randomly selected from the test dataset. A prosthesis state is intended as the combination of locomotion modes before and after the gait event of interest (e.g., from LW to LW, from LW to RD, etc.). Next, the same dataset augmentation techniques adopted for the training dataset are applied to the fine-tune dataset. For the LW-to-LW state (i.e., steady-state level walking), 5 additional samples have been selected to retain the variability of the dominant class. The number of samples was selected to be low enough to be acquired with a very short calibration session; in this case, performing a circuit involving the desired locomotion modes 5 times.

The fine-tuning is a two-step process and the SIAMESE neural network used for it is shown in Fig. 1b. Firstly, the Encoder sub-model is finetuned using the approach described in [26]: an anchor sample from the fine-tune dataset is given as input to the Encoder, whose output is compared via the triplet loss with the output of two samples from the training dataset, one with the same and one with a different state. Secondly, the Classifier is fine-tuned on the fine-tune dataset only. Lastly, the fine-tuned DANN (DANN FT) is recompiled using the fine-tuned Encoder and Classifier sub-models.

### C. Performed Tests

The proposed approach is tested on the HS and TO datasets using a leave-one-session-out approach: one session is used for testing and the others for training. The testing protocol is repeated 5 times. The results of the performed tests are reported using the median f1-score and interquartile range (IQR). The Wilcoxon signed rank test (WSRT) was performed to compare different conditions. The proposed approach is compared with a basic neural network composed of the Encoder, Decoder, and Classifier sub-model (VAE network). The models have been implemented in Keras v.3.4.1. Tests have been performed on a server (256 GB RAM, NVIDIA RTX A6000) using custom Python scripts (Python v.3.9.19).

During the *pretraining phase*, the DANN-VAE network is trained with a batch of 256 for 30 epochs, using the AdamW optimizer [27] with a learning rate of 0.0001 and an exponential decay rate for the 1^st^-moment estimates of 0.5. To promote training stability a warmup approach [28] has been adopted for the learning rate, starting from 0.00001 and reaching 0.0001 after 3 epochs.

During the *fine-tuning phase*, the Encoder is fine-tuned with a batch of 32 samples for 10 epochs, using the Adam optimizer with a learning rate of 0.0001 and an exponential decay rate for the 1^st^-moment estimates of 0.5. The Classifier is finetuned for 10 epochs, using a 32-sample batch and the Adam optimizer with a learning rate of 0.00001 and an exponential decay rate for the 1^st^-moment estimates of 0.5.

The VAE network is trained for 50 epochs using data from a single prosthesis model, with a batch of 256. We used the Adam optimizer with a learning rate of 0.0001 and an exponential decay rate for the 1^st^-moment estimates of 0.5.

Notably, the prostheses used the same impedance parameters for the LW and RA activities. Therefore, despite the networks being trained on all the locomotion modes, it is possible to merge the LW and RA classes to improve the classification accuracy, without impacting the devices’ usability [29]. Consequently, we present the results both with and without LW and RA classes merged.

## III. Results

**Fig. 2.**
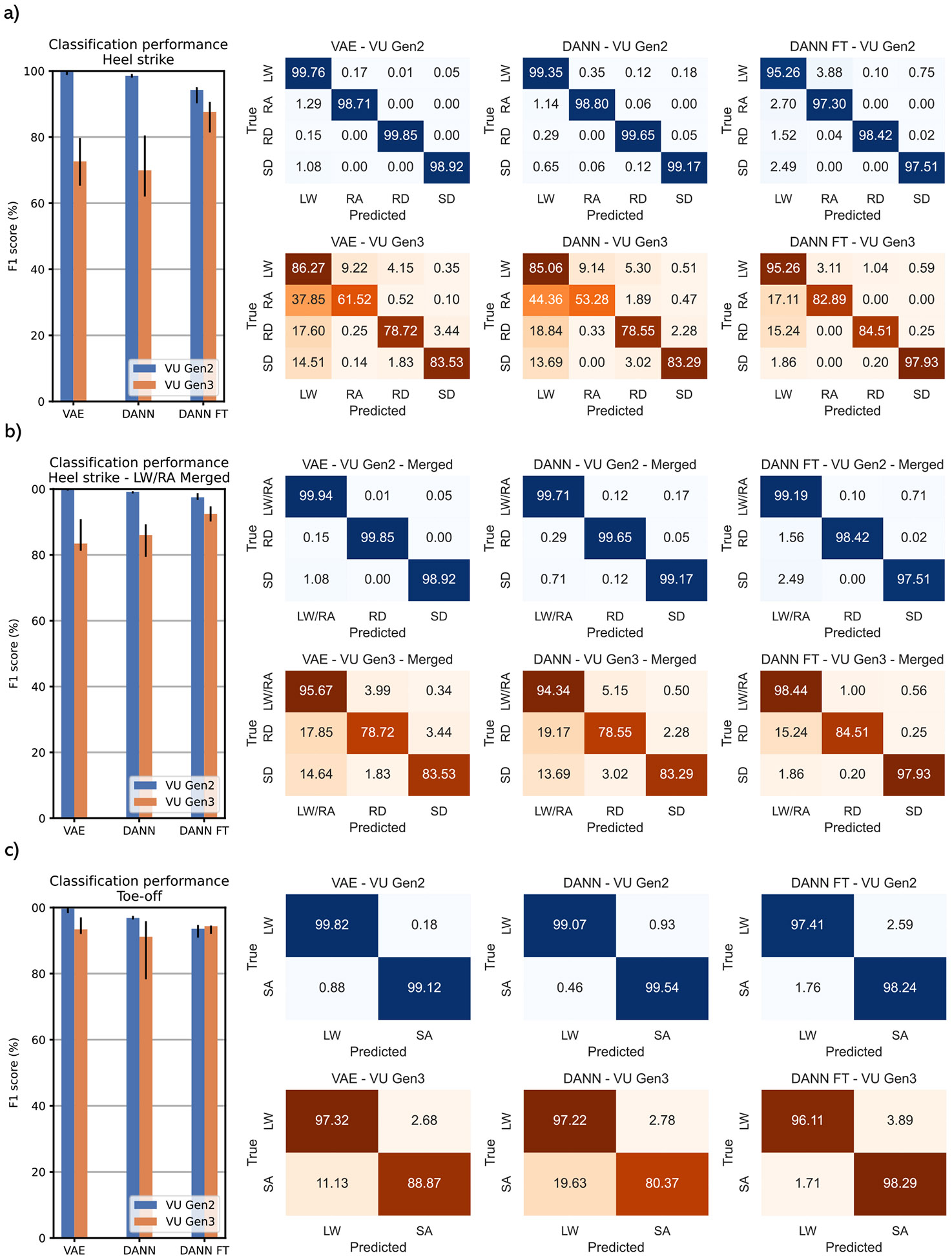
Performance of VAE, DANN, and DANN FT on the HS dataset without merging LW and RA classes (a); the HS dataset merging LW and RA classes (b); the TO dataset (c). The bar plots show the median performance (F1-score, IQR) across the population. The confusion matrixes show the global accuracy on the population (i.e., each matrix is obtained by summing the population confusion matrixes and dividing by the total number of steps of the class).

**Fig. 3.**
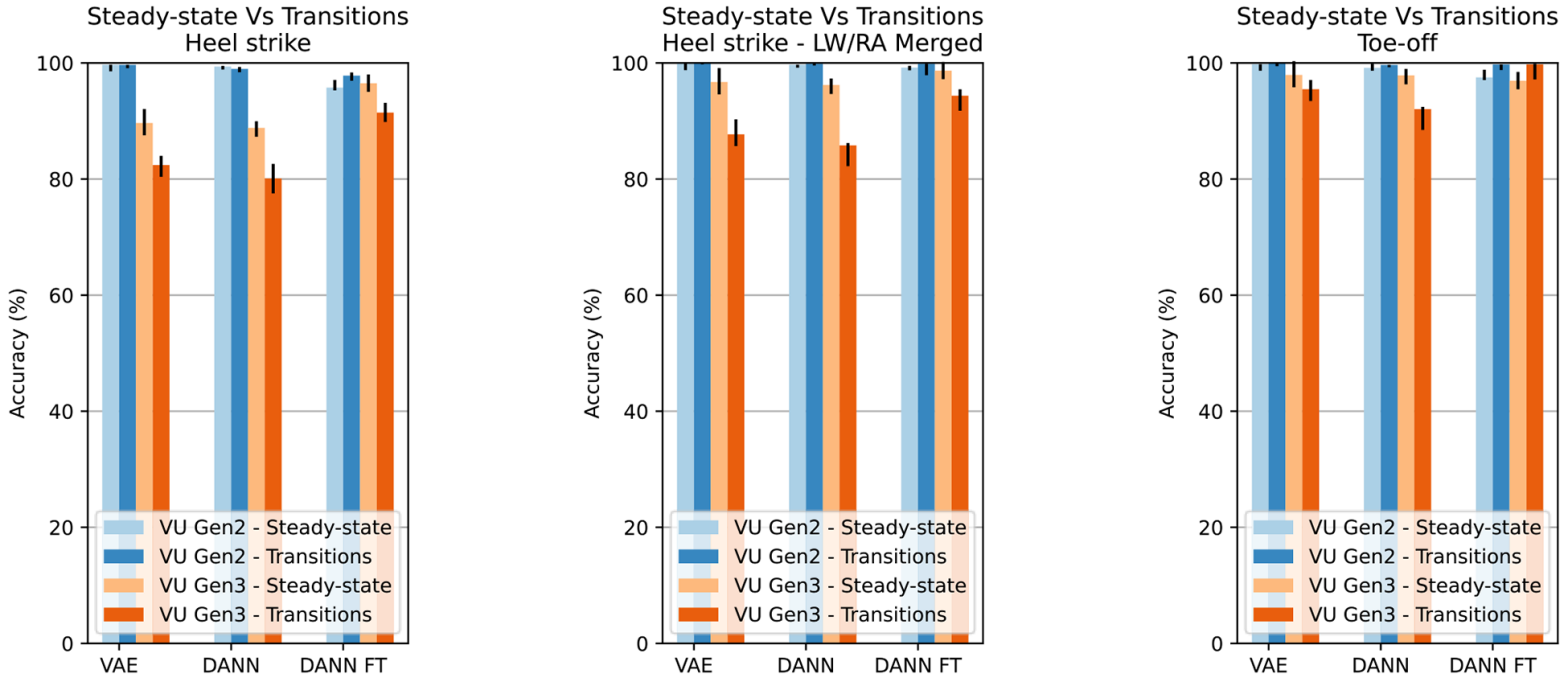
Accuracy (median, IQR) of VAE, DANN, and DANN FT networks on steady-state and transition steps.

The accuracies of both the proposed and comparison approaches are different between prosthesis models, with lower accuracy and increased IQR with the VU Gen 3 (WSRT, p < 0.05) (Fig. 2). Additionally, on VU Gen 2 data, the tested models resulted in similar performances on steady-state and transition steps (WSRT, p < 0.05), while with VU Gen 3, the accuracy on transition steps is lower (WSRT, p < 0.05), except for DANN FT on TO dataset (Fig. 3).

Regarding the *VU Gen 2 results* on the HS dataset, if LW and RA classes are kept separated, VAE, DANN, and DANN FT reach median f1-scores above 94% (Fig. 2a, left). VAE and DANN show similar per-class accuracy, while DANN-FT shows a drop in classification performances, especially in the LW class (Figure 2a, right). If the LW and RA classes are merged (Fig. 2b, left), the f1-score of VAE, DANN, and DANN FT networks is above 96%, with the best performer being the VAE with 99.53% (WSRT, p < 0.05). The TO dataset results in a similar trend, with VAE, DANN, and DANN FT reaching median f1-scores above 94% (Fig. 2c, left) and VAE being the best performer with a median f1-score of 99.64% (WSRT, p < 0.05). The confusion matrixes show similar perclass accuracy between VAE and DANN (WSRT, p > 0.05), while DANN-FT shows a drop in classification performances (Fig. 2c, right).

Regarding the *VU Gen 3 results* on the HS dataset, the VAE and DANN approaches show no statistically significant differences in accuracy with and without LW/RA class merging (WSRT, p > 0.05). Median accuracies are above 70% with LW and RA separated and 83% with LW and RA merged. The DANN FT reaches a median accuracy of 87.63% without class merging and 92.41% with merging, overperforming both VAE and DANN networks (WSRT, p < 0.05) (Fig. 2a, b, left). In general, on VU Gen 3 data, all the proposed networks tend to misclassify RA, RD, and SD classes with LW. The issue is particularly prominent in the RA class. The per-class performance increases noticeably when adopting fine-tuning, especially in the SD class, where the accuracy reaches 97.93% (Fig. 2a, b, right). On the TO dataset, VAE, DANN, and DANN FT show no statistically significant differences in performances (WSRT, p > 0.05), reaching 93.40%, 91.14%, and 94.36% median f1-scores respectively (Fig. 2c, left). It is important to point out that DANN FT presents noticeably improved performances in classifying the SA class, reaching a 98.29% accuracy against 88.87% of VAE and 80.37% of DANN (Fig. 2c, right).

## IV. Discussion

We developed and tested a DL approach using DG and FSL to classify locomotion intent across previously unseen acquisition sessions (or subjects) and multiple prosthesis models. This inter-session classification is crucial for clinical implementation, as users don and doff the prosthesis at least daily. Reducing the amount of data required for algorithm recalibration is essential to reduce the subject’s burden and enhance usability of powered prostheses. Furthermore, the proposed approach’s compatibility with multiple prosthesis models provides a strong foundation for further prosthesisspecific classification models.

Despite their similarity, data from the two prosthesis models present differences which are reflected in the results. At HS, the DANN FT network reached median f1-scores above 92% with VU Gen 3 prosthesis and above 97% with VU Gen 2. At TO, both VAE and DANN networks reach higher median f1-scores on VU Gen 2 data. HS results are consistent across the VAE, DANN, and DANN FT models on the VU Gen 2 dataset; DANN FT largely outperforms the other two approaches with VU Gen 3 data instead. Similarly, DANN FT shows better accuracy in the SA class on the VU Gen 3 TO dataset, while performances drop slightly on the VU Gen 2 dataset. Such a different behavior may be traced to two factors: data quantity and data variability. The VU Gen 2 HS dataset is three times larger than the VU Gen 3 dataset but presents a comparable amount of transition steps, which are more complex to classify. Fig. 3 suggests that a larger dataset can help achieve higher accuracies on transition steps. Additionally, both HS and TO VU Gen 2 datasets contain multiple sessions per subject, promoting better intersession accuracy since data from the tested subjects are present in the training dataset. Ultimately, session and prosthesisspecific approaches may achieve comparable performances when the dataset is large enough. Specifically, the VU Gen 2 data results suggest that fine-tuning may be avoided when multiple sessions per subject are available in large datasets. As suggested in the literature [18], fine-tuning can noticeably improve global and per-class accuracies when the dataset is smaller and more variable instead, as shown by the results on the HS and TO VU Gen 3 datasets.

The need for a common set of onboard sensors between the different prosthesis models represents the main limitation of the work, preventing the possibility of leveraging additional signals in newer prostheses. Additionally, differences in sensor placement and orientation require a fine inspection of data to ensure coherence and reduce possible errors. In a realtime scenario, signal transformation to match the neural network’s expected input must be considered, potentially adding processing time.

In conclusion, the proposed approach successfully created a common base classifier for multiple prosthesis models, achieving accuracies above 92% and up to 99% on data from unseen sessions or subjects. This classifier can be further adapted via fine-tuning for small or highly variable datasets, enhancing both overall and per-class accuracies with only a limited finetuning dataset. Specifically, the fine-tuning samples used in this work can be acquired by performing a circuit including the target locomotion modes only five times. Additionally, the classifier’s accuracies can be further improved by implementing prosthesis-aware constraints, which limit the possible classification outcomes based on the current prosthesis state.

We will next explore the real-time portability of the proposed approach, by assessing its classification and inference performances online with a powered prosthesis. Additionally, the approach will be tested on other prosthesis models to assess its generalizability.

## Figures and Tables

**TABLE I:** Possible Transitions According to the Gait Event and Current Locomotion Mode

		Locomotion mode				
		*LW*	*SA*	*SD*	*RA*	*RD*
**Gait event**	***HS***	LW, SD, RA, RD		LW, SD	LW, RA	LW, RD
	***TO***	LW, SA				
	***FF***		LW			

**TABLE II:** Dataset Composition

Prosthesis	Total steps	Transitions	Steady-state	LW	RA	RD	SA	SD
***HS, VU Gen 2***	32990	1377	31613	27808	1664	1969		1549
***HS, VU Gen 3***	11780	1562	10218	10061	445	556		718
***TO, VU Gen 2***	43469	7115	36294	40283			3186	
***TO, VU Gen 3***	11051	1612	9434	10282			769	
